# The European Association of Nuclear Medicine (EANM)’s Response to the 2023 European Thyroid Association (ETA) clinical practice guidelines for thyroid nodule management and nuclear medicine: a deliberate oversight?

**DOI:** 10.1007/s00259-023-06571-z

**Published:** 2024-01-16

**Authors:** Michael C. Kreissl, Petra Petranović Ovčariček, Alfredo Campenni, Alexis Vrachimis, Murat Tuncel, Luca Giovanella

**Affiliations:** 1 Division of Nuclear Medicine, Department of Radiology and Nuclear Medicine, University Hospital Magdeburg, Otto-Von-Guericke University, 39120 Magdeburg, Germany; 2 Department of Oncology and Nuclear Medicine, University Hospital Center Sestre Milosrdnice, 10000 Zagreb, Croatia; 3 School of Medicine, University of Zagreb, 10000 Zagreb, Croatia; 4 Department of Biomedical and Dental Sciences and Morpho-Functional Imaging, Unit of Nuclear Medicine, University of Messina, 98100 Messina, Italy; 5 Department of Nuclear Medicine, German Oncology Center, University Hospital of the European University, 4108 Limassol, Cyprus; 6 Department of Nuclear Medicine, Hacettepe University, 06230 Ankara, Turkey; 7 Clinic for Nuclear Medicine, Imaging Institute of Southern Switzerland, Ente Ospedaliero Cantonale, 6500 Bellinzona, Switzerland; 8 Clinic for Nuclear Medicine, University Hospital of Zürich, 8004 Zürich, Switzerland

The European Thyroid Association (ETA) has recently published clinical practice guidelines for thyroid nodule management [1]. This is a promising development aiming towards a streamlined and homogene interdisciplinary approach in the management of thyroid nodules. However, several important aspects of nuclear medicine approaches were not adequately addressed in these guidelines, despite regular exchanges between the EANM and the ETA over the past years.

These guidelines were written by a multidisciplinary team, led by two endocrinologists. In addition to four endocrinologists, the team consisted of one internist/clinical cytologist, one endocrine surgeon, one radiologist, one pathologist, and one clinical biologist. Some nuclear medicine physicians who are members of the ETA and the Austrian Thyroid Association did comment on the draft version of the guidelines, however, none of them was included in the authors’ panel, which sidelined them from participating in the discussion process. The authors admitted that, in hindsight, including more specialists (i.e., with a nuclear medicine background) could have been beneficial. The nuclear medicine physicians’ comments were partially incorporated in the guidelines text but not in the recommendations. In our opinion, there is therefore a substantial gap in these guidelines, given that in many European countries (e.g., Germany, Austria, and Croatia), secondary care after nodule detection is mostly provided by nuclear medicine physicians. To prevent this aspect from being overlooked, the EANM should have been involved in the process, and its endorsement of the guidelines should have been requested.

The guidelines were generated by systematically assessing the literature. The Grading of Recommendations, Assessment, Development, and Evaluation (GRADE) framework was used to grade the quality of evidence and help the authors with drafting their clinical practice recommendations. The evidence was rated on a scale ranging from high, moderate, low, to very low, along with the strength of the examined recommendation. In addition, there were also ‘*ungraded good practice statements*’ in the absence of sufficient data. Consensus was reached by two rounds of voting in a modified Delphi process.

## Pros

The guidelines appropriately emphasised the adoption of TIRADS (Thyroid Imaging and Reporting Data System) as a pivotal tool for the risk stratification of thyroid nodules, underscoring its significance for the field. However, colleagues from the ETA considered EU-TIRADS to be the preferred system. This is understandable as ETA experts developed EU-TIRADS. However, this system has yet to be shown to be superior to other TIRADS. On the contrary, in a large-scale German study, EU-TIRADS proved to be inferior [2]. Furthermore, a recent meta-analysis demonstrated that the American College of Radiology Thyroid Imaging Reporting and Data System (ACR TIRADS) has a higher diagnostic performance than EU-TIRADS [3]. Additionally, some authors challenged the role of TIRADS in reducing the number of inappropriate fine-needle aspiration cytology (FNAC) in clinical practice [4]. A recent large-scale Turkish study compared five ultrasound thyroid reporting systems—American Thyroid Association (ATA) guidelines, ACR TIRADS, EU-TIRADS, Korean TIRADS, and American Association Clinical Endocrinologists/American College of Endocrinologists and Associazione Medici Endocrinologi guidelines for the ability to differentiate benign from malignant nodules and to spare patients from unnecessary FNAC [5]. The authors demonstrated that ATA guidelines have the highest area under the curve among all reporting systems and low false negative rates. Even the article cited by the authors [6] concluded that the ACR TIRADS outperformed the other sonographic classification systems, classifying more than half the biopsies as unnecessary with a false negative rate of FNR of 2.2%. Furthermore, in reference to article 51 [7], a significant difference in the choice of TI-RADS preferences among disciplines was reported, with ACR-TIRADS being the first choice for radiologists and nuclear medicine physicians. This also demonstrates the importance of including multidisciplinary panel members to reflect on the different aspects of such controversial issue.

In implementing such systems, caution is needed, especially in non-specialised centers.

## Cons

Given the absence of a nuclear medicine expert in the authors’ panel, it is unsurprising that nuclear medicine diagnostics are only briefly touched upon. Again, as also seen in the ATA guidelines [8], the recommendation only provides guidance on how to perform a thyroid scan if the TSH is subnormal. Only in the background text is it mentioned that hyperfunctioning nodules can be seen with a normal TSH level in countries with previous or current iodine deficiency. In Germany, the largest population in the European Union, 20% of all thyroid nodules are hyperfunctioning, and 80% are found in the setting of a normal TSH [9]. Even if the rate of normal TSH values in hyperfunctioning nodules is lower in other countries with better iodine supply, it can still be expected to be significant [10, 11]. This, in turn, may lead to unnecessary diagnostic procedures, e.g., FNAC and even surgery, considering that a significant percentage of hyperfunctioning nodules have high-risk features on ultrasound and present with a high TIRADS score [12].

Concerning the use of [^99m^Tc]Tc-MIBI and [^18^F]FDG, additions to the text of the guidelines were made, but again, no specific recommendations were formulated. Given the abundance of data on using [^99m^Tc]Tc-MIBI for evaluating thyroid nodules indicating a crucial clinical value, neglect has to be postulated. Indeed, malignancy can be ruled out for indeterminate thyroid nodules classified as hypointense at visual evaluation (i.e., qualitative analysis) of [^99m^Tc]Tc-MIBI scintigraphy with up to 99% negative predictive value [13, 14]. Conversely, a semiquantitative approach using the so-called Wash-out index method (WOind) has been demonstrated to improve the specificity and positive predictive values in patients with indeterminate thyroid nodules classified as iso-hyperintense at [^99m^Tc]Tc-MIBI scintigraphy, i.e. molecular imaging [14, 15]*.* Accordingly, molecular imaging is regularly used in many European countries to reduce unnecessary thyroid surgeries [16].

Recent data from large-scale randomised Dutch trials are available concerning the use of [^18^F]FDG PET/CT, indicating good efficacy and cost-effectiveness in nodules with indeterminate cytology [17, 18].

When it comes to the therapeutic part of the management of thyroid nodules, radioiodine therapy is mentioned as an optional treatment modality. Unfortunately, the description is relatively coarse, and the success rate needs to be mentioned (just the volume reduction, which is less relevant as the second aim of radioiodine therapy in hyperthyroid patients). A reference to existing nuclear medicine guidelines could have been made (ATA) [19].

Against all odds, radioiodine is mentioned as an alternative to surgery and minimally invasive Interventions (MITs) in hyperfunctioning nodules. Here, data on treating hyperfunctioning nodules using MIT are still scarce. So far, MIT, on long-term follow-up, appears inferior [20]. MITs are relatively new in this field. They have yet to fulfill the product life cycle as radioiodine did, and they have not been extensively tested compared to alternatives. Therefore, MIT might be the alternative, not radioiodine!

To our surprise, the guidelines only briefly mention the medical treatment of goiter. The authors recommend not using iodine in replete populations; however, most of the nodules are found in countries with an insufficient supply of iodine. In conclusion, respective recommendations still need to be included.

Moreover, according to the authors, the use of levothyroxine is not recommended because it is not effective. Only one publication from 1998 using TSH-suppressive doses of levothyroxine is mentioned [21]. Other studies could have been examined to support this argument, especially those using non-suppressive doses or combinations with iodine [22].

Finally, the ETA guidelines suggest using rhTSH in non-toxic goiters with low iodine uptake. This refers to a ‘modified release’ rhTSH, given at a much lower dose than the one used in thyroid cancer [23]. It should be noted that this drug is not commercially available, and that this application of rhTSH is out of label. Again, if ‘conventional’ rhTSH is used to stimulate a patient with a thyroid still present, this will result in severe hyperthyroidism and volume increase with compression of surrounding structures.

## Conclusion

The ETA Practical Guidelines were developed by different specialists, but no nuclear medicine physician. Whether this was just a random oversight remains to be determined. However, what is undeniable is the marginalization of nuclear medicine and, above all, the surprising downgrading of radioiodine (and surgery) to MITs alternative despite more than 80 years of successful application.

Whatever the reasons for this lack of engagement with nuclear medicine specialists and nuclear medicine in general, the result is an unbalanced and biased document that, despite what has been stated, is not adequately supported by evidence in some sections.
